# Malnutrition and bilateral central retinal vein occlusion in a young woman: a case report

**DOI:** 10.1186/1752-1947-2-77

**Published:** 2008-03-10

**Authors:** Mark Taubert, Timothy C Dowd, Angela Wood

**Affiliations:** 1 Specialist Registrar in Palliative Medicine, Morriston Hospital, Swansea NHS Trust, Swansea, SA6 6NL, UK; 2 Consultant Ophthalmologist, Department of Ophthalmology, James Cook University Hospital, Marton Road, Middlesbrough, TS4 3BW, UK; 3 Consultant Haematologist, Department of Haematology, James Cook University Hospital, Marton Road, Middlesbrough, TS4 3BW, UK

## Abstract

**Introduction:**

Can vitamin B12 and folate deficiency cause central retinal vein occlusion? We conducted a literature search to find out whether nutritional deficiency of vitamin B12 and folate can lead to impaired vision.

**Case presentation:**

The patient in the article presented in an eye-casualty department in the North East of England with gradual painless visual loss over six weeks. She was found to have bilateral central retinal vein occlusion with significant anaemia and vitamin B12 and folate deficiency.

**Conclusion:**

Vitamin B12 and folate deficiency can lead to elevated levels of homocysteine. We found a large amount of published data relating central retinal vein occlusion to elevated homocysteine levels, but there was a lack of conclusive evidence for this association Patients should be asked about their dietary history where a thrombotic event is suspected or confirmed.

## Introduction

The incidence of retinal vein occlusion varies in population based studies from 2 per thousand to 8 per thousand persons[1,2]. Patients who develop central retinal vein occlusion are typically over 65 years of age and it is a common cause of visual morbidity [3]. There is an increased incidence of central retinal vein occlusion in people with conditions such as diabetes mellitus, hypertension, collagen vascular diseases and hyperviscosity syndromes, with smoking and contraceptive pill use being additional risk factors. When young patients develop a central retinal vein occlusion it is important to obtain a detailed nutritional history, as is shown by this case.

## Case presentation

A 26-year-old Caucasian woman was led into the ophthalmology casualty department by her mother. She had suffered gradual and painless visual loss over the previous six weeks. Her visual acuity on a standard Snellen chart was 6/60 on her right eye and 6/36 on her left eye.

She had no other symptoms other than visual loss, occasional headaches and recently increasing breathlessness on exertion. Previously she had had good vision, not requiring correction.

Her social history was that she lived at her parents' house; she was a non-smoker and had recently been on holiday in Cyprus for three weeks. She denied any casual sexual intercourse whilst on holiday and was not taking any oral contraception. She estimated drinking about 20 units of alcohol at weekends with her friends and was a non-smoker.

She was overweight and remarkably pale. Her conjunctivae were mildly icteric and urinalysis revealed 1+ of bilirubin. Blood pressure was 90/45 mmHg and pulse rate was 92 beats per minute. Examination of the chest and abdomen was unremarkable.

Fundal examination had all the features of bilateral central retinal vein occlusion with both deep and superficial haemorrhages involving all four quadrants of the retina on each side, as well as marked optic disc oedema and dilated, tortuous retinal veins. Intraocular pressures were normal.

She was admitted and found to have a haemoglobin of 4.4 g/dl. Mean cell volume was 125 fl. Platelet count, white cell count and erythrocyte sedimentation rate were normal. Bilirubin was 50 umol/l with otherwise normal liver function tests. Serum glucose was 5.6 mmol/l and serum lipids were normal.

Blood film showed a megaloblastic anaemia with nucleated red cells, macrocytosis and hypersegmented neutrophils. Absolute reticulocyte count was not raised. Low levels of folate and vitamin B12 were confirmed on serum testing (folate: 1 ng/ml, vitamin B12: 54 ng/l).

Fluorescein angiography confirmed the clinical picture of non-ischaemic central retinal vein occlusion. Protein C, protein S and antithrombin III levels were normal. There was no resistance to activated protein C and lupus anticoagulant and antiphospholipid antibodies were negative.

On further closer questioning it was found that the patient had not eaten vegetables for several years and lived on a diet involving a processed corn snack, chips and fast food chain meals. She explained that she did not like the taste of vegetables and dairy products.

Malabsorption causes were excluded over the next weeks and she was given folic acid, hydroxycobalamin and iron supplementation. She was referred to a dietitian and advised on a healthier diet. Visual acuity improved to (Snellen chart) 6/12 on the right and 6/12 on the left over the subsequent months. Her haemoglobin levels returned to normal over the subsequent months.

## Discussion

The case describes a young woman with severe anaemia caused by very poor diet. Her visual acuity gradually deteriorated over several weeks and it turned out she had a bilateral central retinal vein occlusion. This is a very rare event in a young patient.

When we searched the literature, we found associations with malabsorption disorders and retinopathy for example in patients with pernicious anaemia [4]. Isolated retinal haemorrhages are a well recognised complication of severe anaemia and there are case reports describing such presentations with folate and vitamin B12 deficiency [5]. There also appears to be evidence for a link between vitamin deficiencies and retinal veno-occlusive disease; both low serum folate and vitamin B12 levels can lead to elevated homocysteine levels [6,7] and in conjunction pose an important theoretical risk factor for the development of central retinal vein occlusion [8]. Moderately elevated levels of homocysteine are already known to be associated with arterial and venous thrombotic events [9]. We found a large amount of published data relating central retinal vein occlusion to elevated homocysteine levels, but there was a lack of conclusive evidence for this association. In young patients a definite link between high homocysteine levels and risk of developing central retinal vein occlusion has not been established [10].

## Conclusion

Our initial history-taking had focussed on smoking, alcohol consumption and foreign travel. We tried in vain to tie these facts together to determine an aetiology, for this atypical case of bilateral central retinal vein occlusion in a woman in this age group. However, it turned out that the important part of the social history was this patient's nutrition and this is a salutary lesson to doctors of the risks of omitting this important detail from history taking.

The authors suggest including a section in each patient's social history asking specifically about dietary habits, whenever a thrombotic event is suspected, in order to identify quickly nutritional extremes. This is once again a reminder that malnutrition is still very much an issue in modern day Britain.

## Competing interests

The author(s) declare that they have no competing interests.

## Authors' contributions

MT, TCD and AW were all involved in the management of the patient. MT wrote the article and did the literature search. TCD and AW revised and edited the final manuscript. All authors read and approved the final manuscript. MT is guarantor for the article.

## Consent

Written consent was obtained from the patient for publication of the study. Written informed consent was obtained from the patient for publication of this Case report. A copy of the written consent is available for review by the Editor-in-Chief of this journal.
